# Structural and Scintillation Properties of Ce^3+^:Gd_3_Al_3_Ga_2_O_12_ Translucent Ceramics Prepared by One-Step Sintering

**DOI:** 10.3390/ma16093373

**Published:** 2023-04-25

**Authors:** Qi You, Hui Lin, Ruijin Hong, Zhaoxia Han, Dawei Zhang, Yuchong Ding

**Affiliations:** 1 Engineering Research Center of Optical Instrument and System, Ministry of Education and Shanghai Key Lab of Modern Optical System, University of Shanghai for Science and Technology, No. 516 Jungong Road, Shanghai 200093, China; 2 Research & Development Center of Material and Equipment, China Electronics Technology Group Corporation No. 26 Research Institute, Chongqing 400060, China

**Keywords:** Ce^3+^:Gd_3_Al_3_Ga_2_O_12_, one-step sintering, X-ray detector, opto-functional ceramics

## Abstract

Cerium-doped gadolinium aluminum gallium garnet (Ce^3+^:Gd_3_Al_3_Ga_2_O_12_, Ce^3+^:GAGG) ceramic is a promising scintillation material. In this study, Ce^3+^:Gd_3_Al_3_Ga_2_O_12_ scintillation ceramics were prepared by the one-step sintering of commercially available Gd_2_O_3_, Al_2_O_3_, Ga_2_O_3_, and CeO_2_ powders in a flowing oxygen atmosphere at 1600 °C by solid-phase reaction sintering. For all the Ce^3+^:Gd_3_Al_3_Ga_2_O_12_ ceramic samples doped with different amounts of Ce^3+^ doping, dense ceramics were obtained. The structure, photoluminescence, and scintillation properties of the Ce^3+^:Gd_3_Al_3_Ga_2_O_12_ ceramics have been investigated. The average grain size of samples sintered at 1600 °C is about 2 μm. The X-ray excitation luminescence peak is around 560 nm, which is consistent with that of Ce^3+^:Gd_3_Al_3_Ga_2_O_12_ single crystals, matching well with the computed tomography X-ray detector’s response sensitivity. The light yield is higher compared to the standard reference sample—lutetium yttrium orthosilicate single crystal.

## 1. Introduction

Scintillators are light-emitting materials that absorb high-energy particles or rays such as X-ray, γ-ray, and neutrons, and emit near-UV or visible photons [1]. Scintillation detectors have important applications in many fields, such as nuclear medicine, security, and high-energy physics [2]. As the key component in scintillation detectors, scintillator materials have various forms, such as single crystals [3], glass [4], transparent ceramics [5], plastics [6], and quantum dots [7].

In the past 20 years, scintillator detectors have been widely used in X-ray computed tomography (CT) systems. Currently, Pr:Gd_2_O_2_S (GOS), Ce:(Tb,Lu)_3_Al_5_O_12_ (Gemstone^TM^), and Eu:(Y,Gd)_2_O_3_ (YGO, HiLight^TM^) are the three key scintillator materials commonly used in CT systems [8]. GOS was developed by Hitachi and widely used by medical device manufacturers such as Philips and Siemens. Gemstone^TM^ and HiLight^TM^ were developed by GE and are used in their CT products [9]. Ce^3+^: GAGG is a high-quality scintillator material, which has the advantages of high density (6.7 g/cm^3^), high light yield, short luminescence decay time, and no spontaneous radiation [10]. The effective atomic number of the GAGG crystal is as high as 54.4, and its emission peak is also well matched with the photodetector, making it good for applications in CT [11]. In addition, because Ce:GAGG contains the element Gd, the isotope of Gd has the largest known thermal neutron reaction cross section and is suitable for applications such as high-energy physics quantifiers and neutron detectors [12]. In 2012, Yanagida et al. [3] successfully grew a Ce:Gd_3_Ga_3_Al_2_O_12_ single crystal with a light yield of 46,000 ph/MeV at 662 keV and an energy resolution of about 4.9%. In 2016, Ce^3+^:GAGG single crystalline film scintillators prepared by Jan Bok et al. [13] were used for electron detection in SEM. In 2018, Lim et al. [14] prepared Ce^3+^:GAGG scintillation crystals for synchrotron X-ray radiography (SXR). In 2020, Ce^3+^:GAGG scintillator powders were prepared by Gerasymov et al. [15] for the development of X-ray radiography composite detectors.

The cubic crystal structure of GAGG makes it possible to obtain transparent ceramics. The sintering temperature of the ceramics is much lower compared to the growth temperature of single crystals, which largely inhibits the volatilization of gallium oxide. Compared with single-crystal scintillators, transparent ceramic scintillators have the advantages of uniform doping of rare earth ions, high doping concentration, simple and low-cost preparation, and it is easy to prepare large-sized samples [16]. In 2015, Chen et al. [5,9] prepared (Ce,Gd)_3_Al_3_Ga_2_O_12_ ceramics. They investigated the effects of doping with different concentrations of zirconia and different holding times without additives on the optical properties of the samples; however, no further study of the scintillation properties of the samples was carried out. In 2016, Ye et al. [8] prepared Ce^3+^-doped Gd_3_Ga_3_Al_2_O_12_ scintillation ceramics via two steps of sintering: first pre-sintering and then hot isostatic pressing (HIP). HIP plays a key role in increasing the optical transmittance of ceramic samples.

In summary, some of the past studies used two-step sintering, and the additional hot isostatic pressure made the preparation costly. Some of the sintering methods make the sintering temperature higher, which also makes the preparation cost increased. Therefore, the one-step high-temperature solid-phase reaction method used in this paper has good prospects for application.

In this paper, Ce^3+^:GAGG scintillation ceramics were prepared by one-step high-temperature solid-phase reactive sintering in oxygen atmosphere, and the phase composition, crystal structure, microstructure, luminescence spectra, and scintillation decay behavior of the ceramics were investigated.

## 2. Materials and Methods

Ce^3+^:GAGG scintillation ceramics were prepared by high-temperature solid-phase reactive sintering [3]. The purity of the raw material has a great influence on the performance of the ceramics, and the chemical composition of Ce^3+^:Gd_3_Al_3_Ga_2_O_12_ shows the highest light yield [17]. High-purity Gd_2_O_3_ (99.99%, aladdin, Shanghai, China), Al_2_O_3_ (99.99%, aladdin), Ga_2_O_3_ (99.99%, aladdin, Shanghai, China), CeO_2_ (99.99%, aladdin, Shanghai, China), and MgO (99.99%, aladdin, Shanghai, China) commercial powders were used as raw materials and accurately weighed according to the (Ce_x_Gd_1−x_)_3_Al_3_Ga_2_O_12_ (x = 0.0005, 0.002, 0.0035 and 0.005) composition. The mixed powders were prepared by wet ball milling, and the balls were high purity ZrO_2_ balls. The ball-to-material weight ratio was about 3:1. 1 wt% PEG-400 was added as the dispersant, 0.02 wt% MgO and 0.5 wt% tetra-ethyl orthosilicate (TEOS) were added as sintering aids [18]. The mixed powder was ball-milled in anhydrous ethanol at 250 rpm in a planetary ball mill (QM-3SP04, Nanjing Nanda Instrument Co., Ltd., Nanjing, China) for 12 h. After ball milling, the mixed slurry was dried at 100 °C. After drying completely, the powders were granulated through 100 mesh sieves. The obtained powders were calcined at 850 °C in air in a muffle furnace to remove the organic ingredients, then finally the powders for ceramic preparation were obtained. The resulting powders were loaded into a stainless steel mold, uniaxially pressed into Φ20 mm tablets at 10 MPa pressure, and further processed by cold isostatic pressing at 250 MPa. No binder was added during the forming process. In order to suppress the volatilization of Ga_2_O_3_ during sintering, 0.6 L/min of flowing oxygen was introduced into the tube furnace and sintered at 1600 °C [19]. The sintering temperature schedule goes as follows: rising from room temperature to 1100 °C at a rate of 5 °C/min and holding for 15 min; from 1100 °C to 1600 °C at a temperature rise rate of 3 °C/min and then holding for 300 min; reducing the temperature from 1600 °C to 1500 °C at a cooling rate of 3 °C/min and then naturally cooling down to room temperature. The oxygen flow was stopped when the temperature was below 500 °C. Finally, the samples were polished on both sides.

The phase composition and crystal structure of ceramic samples were investigated using an X-ray diffractometer (XRD, Rigaku, MiniFlex 600 type, Tokyo, Japan) equipped with Cu K_α_ radiation in the 2θ range of 10–90° and a scanning step of 0.02°. The grain size and morphology of the ceramic samples were characterized by scanning electron microscopy (SEM, Quanta FEG 250, Washington, DC, USA). The optical absorption spectra were measured at room temperature using a UV-Vis-NIR spectrophotometer (SolidSpec-3700i/3700i, Shimadzu, Kyoto, Japan). Photoluminescence (PL) spectra, photoluminescence excitation (PLE) spectra, temperature-dependent PL spectra, and PL decay curves were measured using a fluorescence spectrometer (FLS-1000, Edinburgh, UK). The X-ray source for X-ray excitation luminescence (XEL) spectroscopy was an X-ray tube (F30III-2) equipped with a tungsten target and a maximum output power of 108 W (72 kV, 1.5 mA). The light yield of the samples was tested using a test system consisting of a ^137^Cs gamma ray source, CR173 photomultiplier manufactured by Beijing Hamamatsu Photon Techniques INC, and a multi-channel analyzer with silicone gel coupling between the photomultiplier and the ceramic.

## 3. Results and Discussion

**Figure 1:**
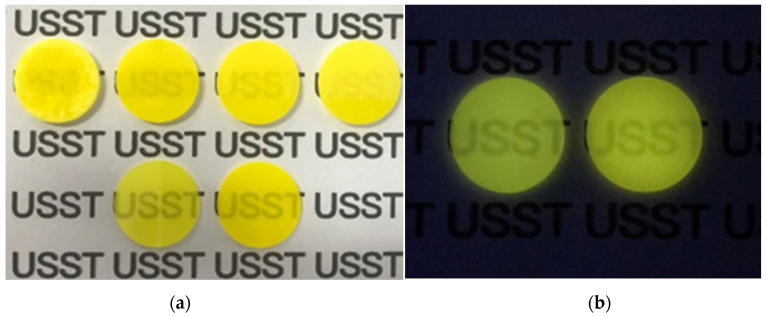
(**a**) First row: polished 0.5%Ce^3+^:GAGG ceramics with different sintering temperatures from 1550 °C to 1650 °C. Second row: 0.05%Ce^3+^:GAGG and 0.5%Ce^3+^:GAGG ceramic samples polished to 0.1 cm thick; (**b**) 0.05%Ce^3+^:GAGG ceramic and 0.5%Ce^3+^:GAGG ceramic samples sintered at 1600 °C under UV lamp irradiation.

The images of the polished Ce^3+^:GAGG ceramics with different sintering temperatures and different concentrations are shown in Figure 1a. All samples are of a diameter of 1.5 cm and a thickness of 0.1 cm. The text underneath the sample is visible, and the color of the sample gradually changes from light yellow to yellow with increasing Ce^3+^ doping concentration. Under the excitation of UV lamp (365 nm) irradiation, the samples emitted strong yellowish green luminescence, as shown in Figure 1b. The SEM, optical absorption spectra, and PLE and PL spectra are discussed later with a 0.05%Ce^3+^:GAGG ceramic sample as an example. The sintering temperatures of each sample in Figure 1a are shown in Table 1.

**Figure 2:**
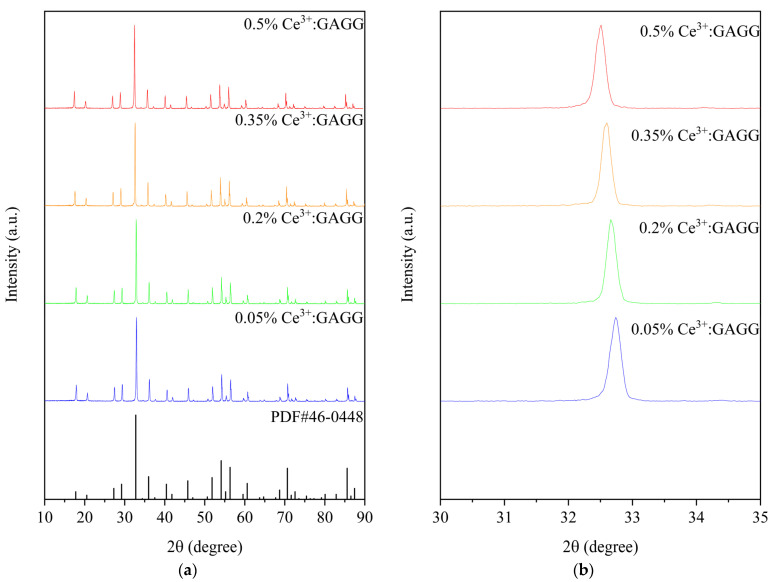
(**a**) XRD patterns of the (Ce_x_Gd_1−x_)_3_Al_3_Ga_2_O_12_ (x = 0.0005, 0.002, 0.0035, and 0.005). (**b**) Enlarged view of the 2θ diffraction peak between 30^°^ and 35°.

**Figure 3:**
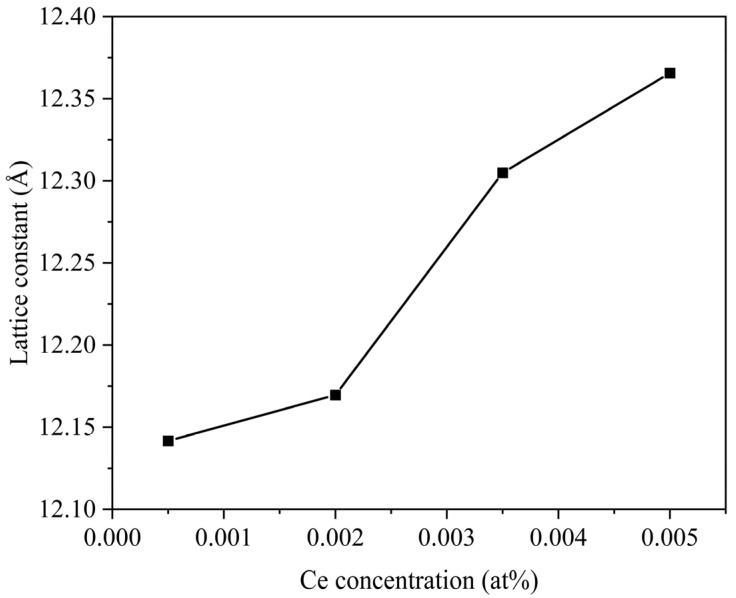
The calculated lattice parameters of the (Ce_x_Gd_1−x_)_3_Al_3_Ga_2_O_12_ (x = 0.0005, 0.002, 0.0035, and 0.005) ceramic samples.

The XRD patterns of (Ce_x_Gd_1−x_)_3_Al_3_Ga_2_O_12_ (x = 0.0005, 0.002, 0.0035, and 0.005) ceramics sintered at 1600 °C are shown in Figure 2a. All the Ce^3+^:GAGG ceramic samples from 10° to 90° diffraction peaks can match the standard card of Gd_3_Al_3_Ga_2_O_12_ (No. #46-0448) well, and no other impurity peaks were detected, which indicates that the pure-phase Ce^3+^:GAGG ceramics have been successfully prepared. As the cerium content increases, the position of the main XRD peaks shifts toward the lower angles, as shown in Figure 2b. Since the ionic radius of Ce^3+^ (102 pm) is larger than that of Gd^3+^ (94 pm), the doping of Ce^3+^ increases the lattice parameters [20]. According to the Bragg formula, the change in the angle of the X-ray diffraction is caused by the change in the crystal plane spacing. The lattice parameters of the samples with different concentrations can be calculated from the XRD patterns. The calculated lattice parameters are shown in Figure 3. The lattice parameters increase with the increase in Ce^3+^ concentration.

**Figure 4:**
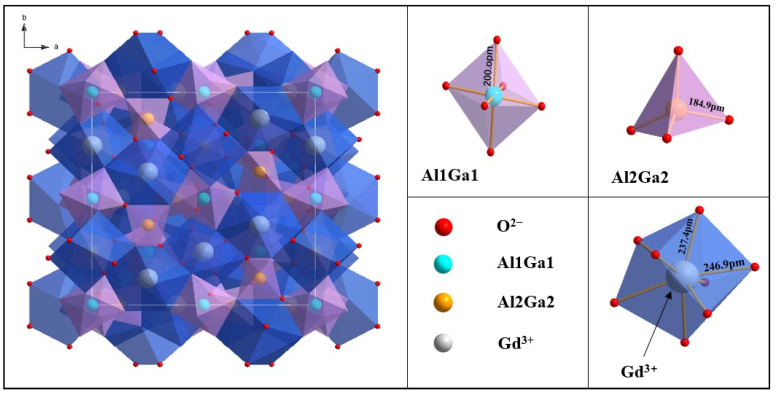
Schematic diagram of the crystal structure of GAGG and the three cationic sites.

The structure of garnet has three cationic sites, that is, the dodecahedral, the octahedral, and the tetrahedral lattices. The crystal structure of GAGG drawn using Diamond^TM^ is shown in Figure 4, where Gd^3+^ occupies the eight-O^2−^-coordinated dodecahedral site, and Al^3+^ and Ga^3+^ occupy the six-O^2−^-coordinated octahedral and four-O^2−^-coordinated tetrahedral sites, respectively. When Ce^3+^ is doped, it generally enters the dodecahedral lattices. GAGG crystals are derived from GGG crystals, which are an excellent host material for laser gain media. Because Al and Ga are the same main group elements and the radius of Al^3+^ is smaller, Al^3+^ makes random disorderly substitutions for Ga^3+^ in the tetrahedral and the octahedral sites; Ga_2_O_3_ is volatile at high temperatures, and the price of Al_2_O_3_ as a raw material is lower, so it is suitable to replace part of Ga^3+^ with Al^3+^. More importantly, the optimization of ceramic properties can be achieved through the regulation of the Ga^3+^ and Al^3+^ ratio [2].

**Figure 5:**
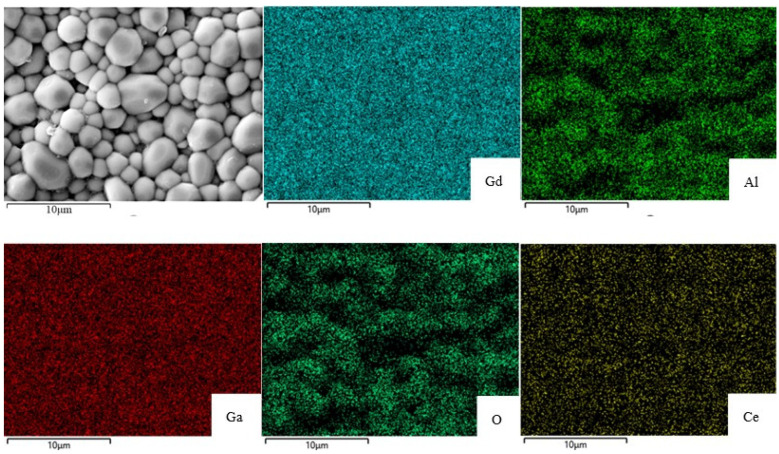
The SEM cross-sectional images and EDS element mapping of 0.05%Ce^3+^:GAGG ceramic sample sintered at 1600 °C.

The removal of residual pores between grains during grain growth is an important step in the process of ceramic sintering to achieve a dense microstructure [21]. The SEM cross-sectional images of the Ce^3+^:GAGG ceramic sample sintered at 1600 °C are shown in Figure 5. The ceramics have a uniform grain size with an average particle size of 2 μm. The fracture surface morphology shows a dense microstructure with no obvious pores or second phases, indicating the absence of impurities. No abnormal grain growth was also observed. Compared with other studies in the literature, where the average grain sizes are around 5 μm or even larger [5,8,9,20], the grain size of ceramics is smaller.

The results of the EDS element mapping of the 0.05%Ce^3+^:GAGG ceramic are shown in Figure 5. The element mapping shows that Gd, Al, Ga, O, and Ce are uniformly distributed throughout the sample, further demonstrating the homogeneous microstructure of the ceramic samples. Mg elements were not detected in the plots due to the small amount of addition.

**Figure 6:**
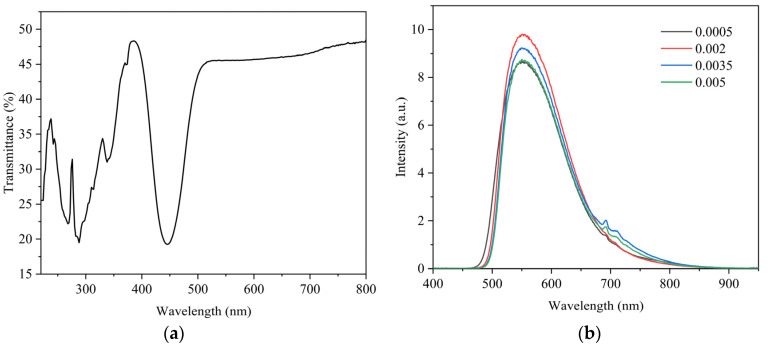
(**a**) Optical transmittance spectrum of 0.05% Ce^3+^:GAGG ceramic sample. (**b**) XEL spectra of Ce^3+^:GAGG ceramics with different concentrations.

The optical transmittance spectra of the 0.05%Ce:GAGG scintillation ceramic measured at room temperature are shown in Figure 6a. The transmittance of the sample at 563 nm is 45.5%, and for scintillation ceramics, if the sample has a high transmittance, the scintillation photons are more easily detected by the diode through the ceramic, resulting in better scintillation performance [22]. The transmission spectrum shows that there are two absorption bands located at 338 nm and 445 nm, which are characteristic absorption bands of Ce^3+^, mainly due to the external electron leap of 4f→5d_2_ and 4f→5d_1_ of Ce^3+^ [23]. In addition to this absorption band, another absorption band is seen between 220 nm and 320 nm. The absorption band here may be formed due to the presence of charge transfer between Ce^4+^ and O^2−^ [24].

The XEL spectra of Ce^3+^:GAGG ceramics with different concentrations are shown in Figure 6b, and they have a broad emission with a peak wavelength at about 560 nm, which is associated with the 5d-4f electron transition of Ce^3+^. The profile of the XEL spectra is consistent with the counterpart of the PL spectrum. This luminescence profile matches the receiving wavelength of the photodiode well, which is more suitable for the CT X-ray detector’s response sensitivity.

**Figure 7:**
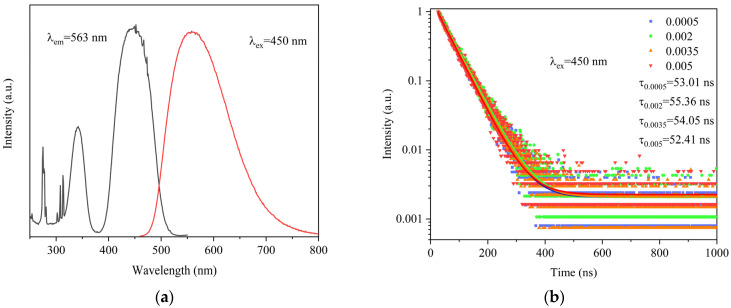
(**a**) PLE (λ_em_ = 563 nm) and PL (λ_ex_ = 450 nm) spectra of 0.05%Ce: GAGG ceramics. (**b**) PL decay curves of Ce^3+^:GAGG ceramics with different concentrations.

The PL and PLE spectra of the 0.05%Ce^3+^:GAGG ceramics are shown in Figure 7a. The spectra show a yellow-green emission band with the peak emission located around 563 nm under light excitation at 450 nm, which is related to the 5d-4f electron transition. The two main excitation peaks are located around 340 nm and 450 nm, which are related to the 4f→5d_2_ and 4f→5d_1_ electron transitions of Ce^3+^, respectively. In addition, the excitation peaks located at 275 nm, 308 nm, and 313 nm are due to the Gd^3+^ 4f-4f electron transitions, which also confirms the existence of energy transfer between Gd^3+^ and Ce^3+^. When the 4f-5d_1_ electron transition is monitored at 450 nm, the emission band is located between 500 nm and 650 nm, which is due to the electron transition of Ce^3+^ from 5d level to 4f level [25].

The PL decay curves of (Ce_x_Gd_1−x_)_3_Al_3_Ga_2_O_12_ (x = 0.0005, 0.002, 0.0035, and 0.005) measured at 450 nm excitation at room temperature are shown in Figure 7b. All the decay curves can be well fitted using a single exponential function as follows:

$$I\left(t\right)=A_{1}exp\left(-t/t_{1}\right)+I_{0}$$

where *I*(*t*) is the luminescence intensity at time *t*, I_0_ is the initial luminescence intensity, *A*_1_ is a constant, *t* is the time, and *t*_1_ is the luminescence decay time. The fluorescence lifetime values of Ce^3+^ in all ceramic samples were 53.01, 55.36, 54.05, and 52.41 ns, respectively.

**Figure 8:**
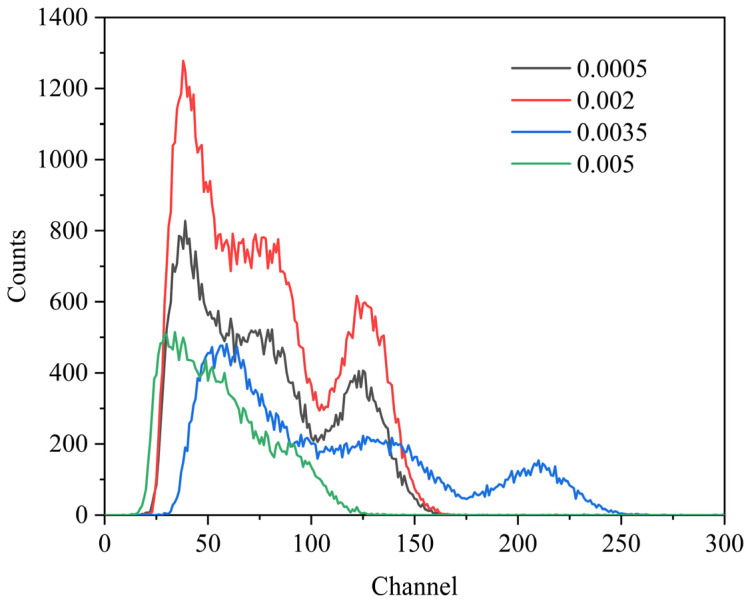
^137^Cs pulse height spectrum of Ce^3+^:GAGG ceramics with different concentrations of Ce^3+^.

The ^137^Cs pulse height spectrum of Ce^3+^:GAGG ceramics is shown in Figure 8 [26,27]. The light yield of the ceramics was calculated by comparing it with the LYSO of known light yield. First, measure the channel number of a standard sample LYSO with known light yield. Then, measure the channel number of the sample to be tested under the same conditions. Since samples with different luminescence wavelengths are matched to the photomultiplier differently, the light yield of the sample to be tested is calculated by the following equation:

$${LY}_{m}=\frac{{LO}_{m}}{{LO}_{s}}\cdot {LY}_{s}\cdot \eta$$

where *LY_m_* is the light yield of Ce^3+^:GAGG to be measured and *LY_s_* is the light yield of the standard reference sample LYSO. *LO_m_* is the number of channels of Ce^3+^:GAGG to be measured and *LO_s_* is the number of channels of the standard reference sample LYSO. *η* is calculated from the ratio of the wavelength of luminescence emission of different materials to the optimum wavelength of the photomultiplier. The light yield of 0.35%Ce^3+^:GAGG was calculated to be 31,500 ph/MeV, which is slightly higher than the light yield of the standard reference sample. It is expected to further increase the light yield of the sample by appropriately increasing the grain size of the sample.

## 4. Conclusions

Pure garnet-phase Ce^3+^:Gd_3_Al_3_Ga_2_O_12_ scintillation ceramics were prepared by a one-step solid-phase reactive sintering in flowing pure oxygen. The luminescence properties of the Ce^3+^:Gd_3_Al_3_Ga_2_O_12_ ceramics, including photoluminescence spectra, photoluminescence decay curves, and X-ray excitation luminescence spectra, were investigated in detail. In the photoluminescence spectra and X-ray excitation luminescence spectra, the luminescence peaks match well with the receiving wavelength of the photodiode. The decay time of Ce^3+^:Gd_3_Al_3_Ga_2_O_12_ scintillation ceramics under 450 nm excitation can be well fitted with a single exponential function. The 50 ms decay time meets the requirements of rapid scintillator decay. The light yield of Ce^3+^:Gd_3_Al_3_Ga_2_O_12_ scintillation ceramics is up to 31,500 ph/MeV, which is better than the standard reference sample lutetium yttrium orthosilicate single crystal, and further improvement by changing the grain size is expected subsequently.

## Figures and Tables

**Table 1:** The sintering temperature of each sample in Figure 1a.

Sample Location	Sintering Holding Temperature
First in the first row	1550 °C
Second in the first row	1575 °C
Third in the first row	1625 °C
Fourth in the first row	1650 °C
Second row	1600 °C

## Data Availability

For any data request, please contact the corresponding authors.

## References

[1] Yanagida T. (2018). Inorganic scintillating materials and scintillation detectors. Proc. Jpn. Acad. Ser. B.

[2] Sibczynski P., Iwanowska-Hanke J., Moszyński M., Swiderski L., Szawłowski M., Grodzicka M., Szczęśniak T., Kamada K., Yoshikawa A. (2015). Characterization of GAGG:Ce scintillators with various Al-to-Ga ratio. Nucl. Instrum. Methods Phys. Res. A.

[3] Kamada K., Yanagida T., Endo T., Tsutumi K., Usuki Y., Nikl M., Fujimoto Y., Fukabori A., Yoshikawa A. (2012). 2 inch diameter single crystal growth and scintillation properties of Ce:Gd_3_Al_2_Ga_3_O_12_. J. Cryst. Growth.

[4] Dormenev V., Amelina A., Auffray E., Brinkmann K.-T., Dosovitskiy G., Cova F., Fedorov A., Gundacker S., Kazlou D., Korjik M. (2021). Multipurpose Ce-doped Ba-Gd silica glass scintillator for radiation measurements. Nucl. Inst. Methods Phys. Res. A.

[5] Chen X., Qin H., Zhang Y., Luo Z., Jiang J., Jiang H. (2015). Preparation and Optical Properties of Transparent (Ce,Gd)_3_Al_3_Ga_2_O_12_ Ceramics. J. Am. Ceram. Soc..

[6] Cester D., Nebbia G., Stevanato L., Pino F., Viesti G. (2014). Experimental tests of the new plastic scintillator with pulse shape discrimination capabilities EJ-299-33. Nucl. Instrum. Methods Phys. Res. A.

[7] Park J.M., Kim H.J., Hwang Y.S., Kim D.H., Park H.W. (2014). Scintillation properties of quantum-dot doped styrene based plastic scintillators. J. Lumin..

[8] Ye Y., Liu P., Yan D., Xu X., Zhang J. (2017). Fabrication of Ce^3+^ doped Gd_3_Ga_3_Al_2_O_12_ ceramics by reactive sintering method. Opt. Mater..

[9] Chen X., Qin H., Zhang Y., Jiang J., Jiang H. (2015). Highly transparent ZrO_2_-doped (Ce,Gd)_3_Al_3_Ga_2_O_12_ ceramics prepared via oxygen sintering. J. Eur. Ceram. Soc..

[10] Babin V., Bohacek P., Grigorjeva L., Kucera M., Nikl M., Zazubovich S., Zolotarjovs A. (2017). Effect of Mg^2+^ ions co-doping on luminescence and defects formation processes in Gd_3_(Ga,Al)_5_O_12_:Ce single crystals. Opt. Mater..

[11] McDonald K., Schweitzer G. (2018). Synthesis of GAGG:Ce^3+^ powder for ceramics using mechanochemical and solution combustion methods. J. Am. Ceram. Soc..

[12] Wang Z., Guo H., Qian S., Zhu Y., Hu P., Wu Q., Chen P., Ma L., Peng S., Zhang L. (2020). Performance study of GAGG:Ce scintillator for gamma and neutron detection. J. Instrum..

[13] Bok J., Lalinský O., Hanuš M., Onderišinová Z., Kelar J., Kučera M. (2016). GAGG: Ce single crystalline films: New perspective scintillators for electron detection in SEM. Ultramicroscopy.

[14] Lim J., Park K., Kim H., So J., Kim J. (2019). Potential of GAGG:Ce scintillation crystals for synchrotron X-Ray micro-imaging. Current Appl. Phys..

[15] Gerasimov I., Nepokupnaya T., Boyarintsev A., Sidletskiy O., Kurtsev D., Voloshyna O., Trubaieva O., Boyarintseva Y., Sibilieva T., Shaposhnyk A. (2020). GAGG: Ce composite scintillator for X-ray imaging. Opt. Mater..

[16] Mori M., Xu J., Okada G., Yanagida T., Ueda J., Tanabe S. (2016). Comparative study of optical and scintillation properties of Ce:YAGG, Ce:GAGG and Ce:LuAGG transparent ceramics. J. Ceram. Soc. Jpn..

[17] Chen X., Qin H., Zhang Y., Jiang J., Wu Y., Jiang H. (2017). Effects of Ga substitution for Al on the fabrication and optical properties of transparent Ce:GAGG-based ceramics. J. Eur. Ceram. Soc..

[18] Mohammadi F., Mirzaee O., Tajally M. (2018). Influence of TEOS and MgO addition on slurry rheological, optical, and microstructure properties of YAG transparent ceramic. Opt. Mater..

[19] Kamada K., Kurosawa S., Prusa P., Nikl M., Kochurikhin V., Endo T., Tsutumi K., Sato H., Yokota Y., Sugiyama K. (2014). Cz grown 2-in. size Ce:Gd_3_(Al,Ga)_5_O_12_ single crystal; relationship between Al, Ga site occupancy and scintillation properties. Opt. Mater..

[20] Chen X., Qin H., Zhang Y., Liu Y., Jiang J., Jiang H. (2016). Microstructure and optical properties of transparent Nd:GAGG ceramics prepared via solid-state reactive sintering. Opt. Mater. Express.

[21] Lee S.H., Kochawattana S., Messing G.L., Dumm J.Q., Quarles G., Castillo V. (2006). Solid-State Reactive Sintering of Transparent Polycrystalline Nd: YAG Ceramics. J. Am. Ceram. Soc..

[22] Xu J., Shi Y., Xie J., Lei F. (2013). Fabrication, Microstructure, and Luminescent Properties of Ce^3+^—Doped Lu_3_Al_5_O_12_ (Ce:LuAG) Transparent Ceramics by Low-Temperature Vacuum Sintering. J. Am. Ceram. Soc..

[23] Liu X., Li H., Xie R., Hirosaki N., Xu X., Huang L. (2006). Cerium-doped lutetium aluminum garnet optically transparent ceramics fabricated by a sol-gel combustion process. J. Mater. Res..

[24] Nikl M., Kamada K., Babin V., Pejchal J., Pilarova K., Mihokova E., Yoshikawa A. (2014). Defect engineering in Ce-doped aluminum garnet single crystal scintillators. Cryst. Growth Des..

[25] Chen X., Hu Z., Feng Y., Liu X., Chen H., Shi Y., Kucerkova R., Beitlerova A., Nikl M., Li J. (2019). Luminescence and scintillation characteristics of cerium doped Gd_2_YGa_3_Al_2_O_12_ ceramics. Opt. Mater..

[26] Zhu Y., Qian S., Wang Z., Guo H., Ma L., Wang Z., Wu Q. (2020). Scintillation properties of GAGG:Ce ceramic and single crystal. Opt. Mater..

[27] Taggart M., Nakhostin M., Sellin P. (2019). Investigation into the potential of GAGG:Ce as a neutron detector. Nucl. Inst. Methods Phys. Res. A.

